# Novel mutations in *HSF4* cause congenital cataracts in Chinese families

**DOI:** 10.1186/s12881-018-0636-3

**Published:** 2018-08-24

**Authors:** Zongfu Cao, Yihua Zhu, Lijuan Liu, Shuangqing Wu, Bing Liu, Jianfu Zhuang, Yi Tong, Xiaole Chen, Yongqing Xie, Kaimei Nie, Cailing Lu, Xu Ma, Juhua Yang

**Affiliations:** 1 Biomedical Engineering Center, Fujian Medical University, Fuzhou, Fujian China; 2 Graduate School of Peking Union Medical College, Beijing, China; 3 National Center for Human Genetics, Beijing, China; 4 National Human Genetic Resources Center, National Research Institute for Family Planning, Peking Union Medical College, 12 Da-hui-si, Hai Dian, Beijing, 100081 China; 5 Department of Ophthalmology, the First Affiliated Hospital of Fujian Medical University, Fuzhou, Fujian China; 6 Fuzhou Southeast Eye Hospital, Fuzhou, Fujian China; 7 Department of Ophthalmology, Hangzhou Red-cross hospital, Zhejiang, Hangzhou China; 8 Xiamen Eye Center of Xiamen University, Xiamen, Fujian China

**Keywords:** Congenital cataracts, Mutation, *HSF4*, Chinese

## Abstract

**Background:**

Congenital cataract, a kind of cataract presenting at birth or during early childhood, is a leading cause of childhood blindness. To date, more than 30 genes on different chromosomes are known to cause this disorder. This study aimed to identify the *HSF4* mutations in a cohort from Chinese families affected with congenital cataracts.

**Methods:**

Forty-two unrelated non-syndromic congenital cataract families and 112 ethnically matched controls from southeast China were recruited from the southeast of China. We employed Sanger sequencing method to discover the variants. To confirm the novel mutations, STR haplotypes were constructed to check the co-segregation with congenital cataract. The pathogenic potential of the novel mutations were assessed using bioinformatics tools including SIFT, Polyphen2, and Human Splicing Finder. The pathogenicity of all the mutations was evaluated by the guidelines of American College of Medical Genetics and InterVar software.

**Results:**

No previously reported *HSF4* mutations were found in all the congenital cataract families. Five novel *HSF4* mutations including c.187 T > C (p.Phe63Leu), c.218G > T (p.Arg73Leu), c.233A > G (p.Tyr78Cys), IVS5 c.233-1G > A and c.314G > C (p.Ser105Thr) were identified in five unrelated families with congenital cataracts, respectively. These mutations co-segregated with all affected individuals in each family were not observed in the unaffected family members or in 112 unrelated controls. All five mutations were categorized to be the disease “pathogenic” according to ACMG guidelines and using InterVar software. Mutations in the *HSF4* were responsible for 11.90% Chinese families with congenital cataracts in our cohort.

**Conclusions:**

In the study, we identified five novel *HSF4* mutations in Chinese families with congenital cataracts. Our results expand the spectrum of *HSF4* mutations causing congenital cataracts, which may be helpful for the molecular diagnosis of congenital cataracts in the era of precision medicine.

**Electronic supplementary material:**

The online version of this article (10.1186/s12881-018-0636-3) contains supplementary material, which is available to authorized users.

## Background

Congenital cataracts (CC) are a kind of cataracts that present at birth or during early childhood, which account for the most important causes of severe visual impairment in the children especially in infants [1]. More than 1 million childhood blindness is resulted from congenital cataracts in Asia, while around 400,000 of whom probably in China. In the developing countries, 7.4–15.3% of childhood blindness results from congenital cataracts. The prevalence of congenital cataracts in children has been estimated about 1–15/10,000 [2]. Prevention of visual impairment caused by congenital cataracts is an important component of the World Health Organization’s (WHO) international program for the elimination of avoidable blindness by 2020 [3].

Genetic factors play key roles in the development of congenital cataracts. With the genetic heterogeneity of congenital cataracts, more than 30 genes on different chromosomes are known to cause the disorder, which include crystallins genes, lens specific connexins genes, major intrinsic protein or aquaporine genes, cytoskeletal structural proteins genes, paired-like homeodomain transcription factor 3 genes, avian musculoaponeurotic fibrosarcoma, and heat shock transcription factor 4 [4]. The most frequent modes of inheritance are autosomal dominant (AD), autosomal recessive (AR), and then X-linked recessive. Based on our survey in Chinese population, the eighteen genes, containing *CRYAA*, *CRYAB*, *CRYBA1*, *CRYBA4*, *CRYBB1*, *CRYBB2*, *CRYBB3*, *CRYGC*, *CRYGD*, *CRYGS*, *GJA8*, *GJA3*, *HSF4*, *MIP*, *BFSP2*, *EPHA2*, *FYCO1* and *PITX3,* can be chosen as the candidate genes to screen congenital cataracts.

In this study, 42 unrelated families with congenital cataracts were recruited from Southeast China, and five mutations in the *HSF4* (MIM# 602438, heat shock transcription factor 4) cause congenital cataracts were reported.

## Methods

### Subjects and DNA specimens

As part of a genetic screening program for genetic eye disorders, we collected peripheral blood from 42 families with congenital cataracts and 225 related individuals from the southeast China. All the affected individuals and unaffected relatives in their family were performed ophthalmological examinations by slit lamp photography. The study followed the tenets of the Declaration of Helsinki. Informed consents were obtained from each participant except for the children. For any participants that are under the age of 16, the consent to participate was obtained from their parents or legal guardians. 112 samples from ethnically matched control individuals were obtained prior to the study. The experiments were approved by the Ethics Committee of Fujian Medical University. Total genomic DNA was extracted from whole blood using the Wizard Genomic DNA Purification Kit (Promega, Beijing, China) according to the manufacturer’s instructions.

### Mutation screening

Before this study, we had compiled hot-spot regions of cataract-causing mutations. Briefly, 72 mutant exons of 31 pathogenic genes associated with 299 congenital cataract families or sporadic cases have been reported in 210 selected articles. The 72 exons, account for 34.62% of all the 208 exons in the 31 genes, were ordered by the summary frequency of disease-causing mutations decreasingly across each gene exons, and the top 26 exons in 18 pathogenic genes were selected as the hot-spot mutation regions. The hot-spot regions covered about 80 percentages of mutations in the compiled mutations with only 36.11 percentages (26/72) of all the mutant exons, and 12.5 percentages (26/208) of all the exons.

All the mutations in the 18 common genes causing congenital cataracts were screened for all the probands of 42 families. These genes including *CRYAA*, *CRYAB*, *CRYBA1*, *CRYBA4*, *CRYBB1*, *CRYBB2*, *CRYBB3*, *CRYGC*, *CRYGD*, *CRYGS*, *GJA8*, *GJA3*, *HSF4*, *MIP*, *BFSP2*, *EPHA2*, *FYCO1* and *PITX3*. The selected hot spot exons and splice junctions of these genes were amplified by PCR from genomic DNA. The PCR primers and conditions for HSF4 were listed in Table 1, and that for other genes listed in Additional file 1: Table S1. PCR products were purified and directly sequenced on an ABI 3730XL Automated Sequencer (PE Biosystems, Foster City, CA) using the same PCR primers. Intra-familial segregation analysis was performed after identification of *HSF4* mutations in probands. The identified *HSF4* mutations were also checked in 112 normal unrelated individuals from the same ethnic background.

**Table 1:** The PCR primers and conditions for *HSF4* primers

Exon	Primer name	Primer sequence(5′-3′)	Amplicon size (bp)	PCR condition
4, 5	HSF4e4/5F	GGACCCAAGAGTGAGCATGA	481	58 °C/GC Buffer1
	HSF4e4/5R	CCCTCCTCCTCTTTGCTCAT		

### Haplotyping analysis

To validate the co-segregation of the novel mutations, the genotyping was performed with three selected microsatellite markers flanking each corresponding pathogenic gene in available family members. Three microsatellites in *HSF4* include D16S3043, D16S3067 and D16S496. Briefly, PCR products from each DNA sample were separated by gel electrophoresis with a fluoresence-based on ABI 3730 automated sequencer (Applied Biosystems) using ROX-500 as the internal lane size standard. The amplified DNA fragment lengths were assigned to allelic sizes with GeneMarker Version 2.4.0 software (SoftGenetics, State College, Pennsylvania, USA). Cyrillic (version 2.1) software was employed to manage the Pedigree and haplotype data.

### Bioinformatics analysis

Mutations description followed the recommendation of the Human Genomic Variation Society (HGVS). The effects of novel missense mutations on the encoded proteins were further evaluated by Polymorphism Phenotyping [5] v2 (PolyPhen-2) and Sorting Intolerant From Tolerant [6] v5.1.1 (SIFT), and the effects of intronic variants on splicing site changes were predicted by the Human Splicing Finder [7] v2.4.1 (HSF). The pathogenicity of all the mutations was evaluated by the standards and guidelines of American College of Medical Genetics and Genomics [8] (ACMG) using InterVar [9] software.

## Results

### Clinical description

**Fig. 1:**
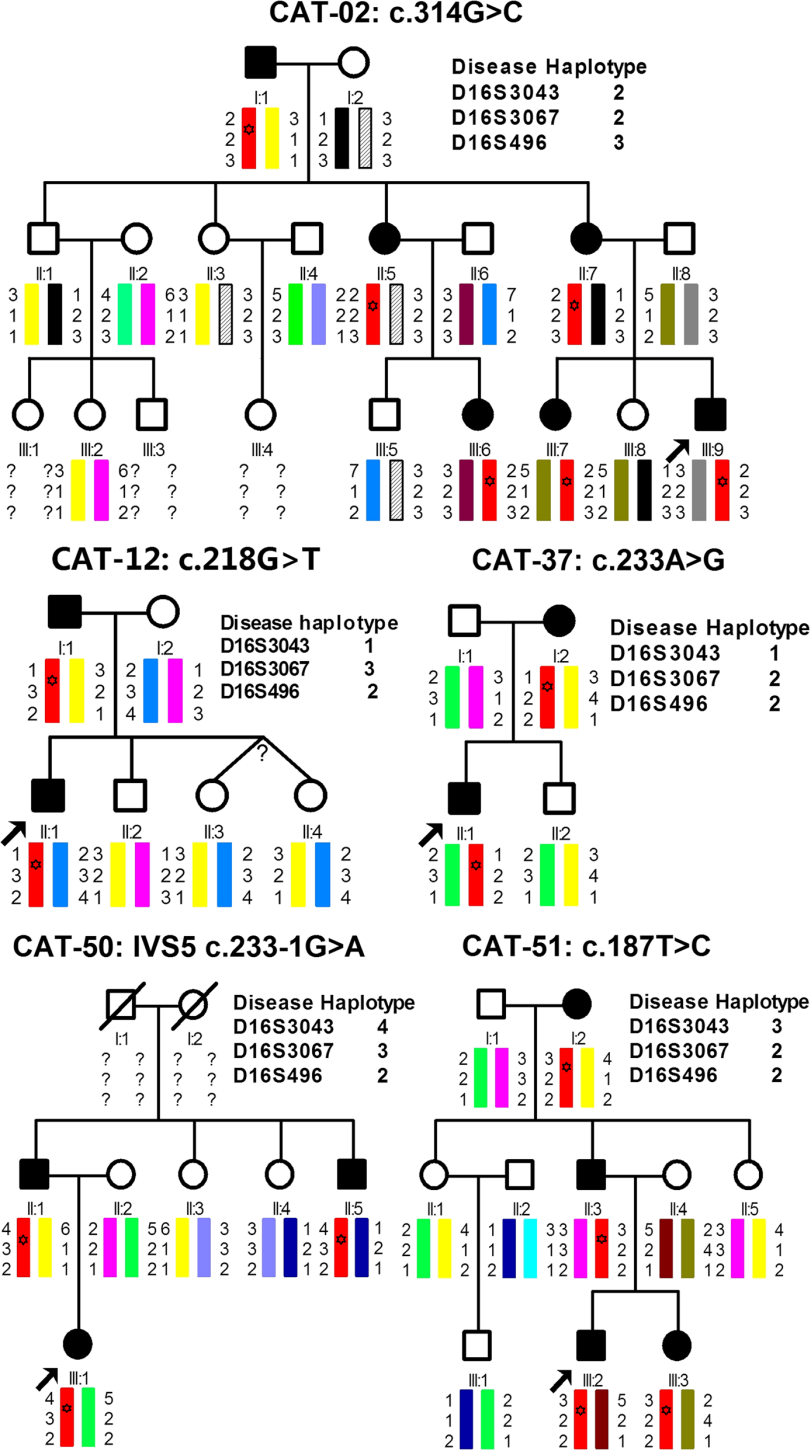
Haplotypes of gene *HSF4* in each family. All the pathogenic haplotypes (red color) indicate that segregation of the haplotypes in affected individuals in each family but not in the unaffected family members. The mutations were labeled with hexagrams in the haplotypes

**Fig. 2:**
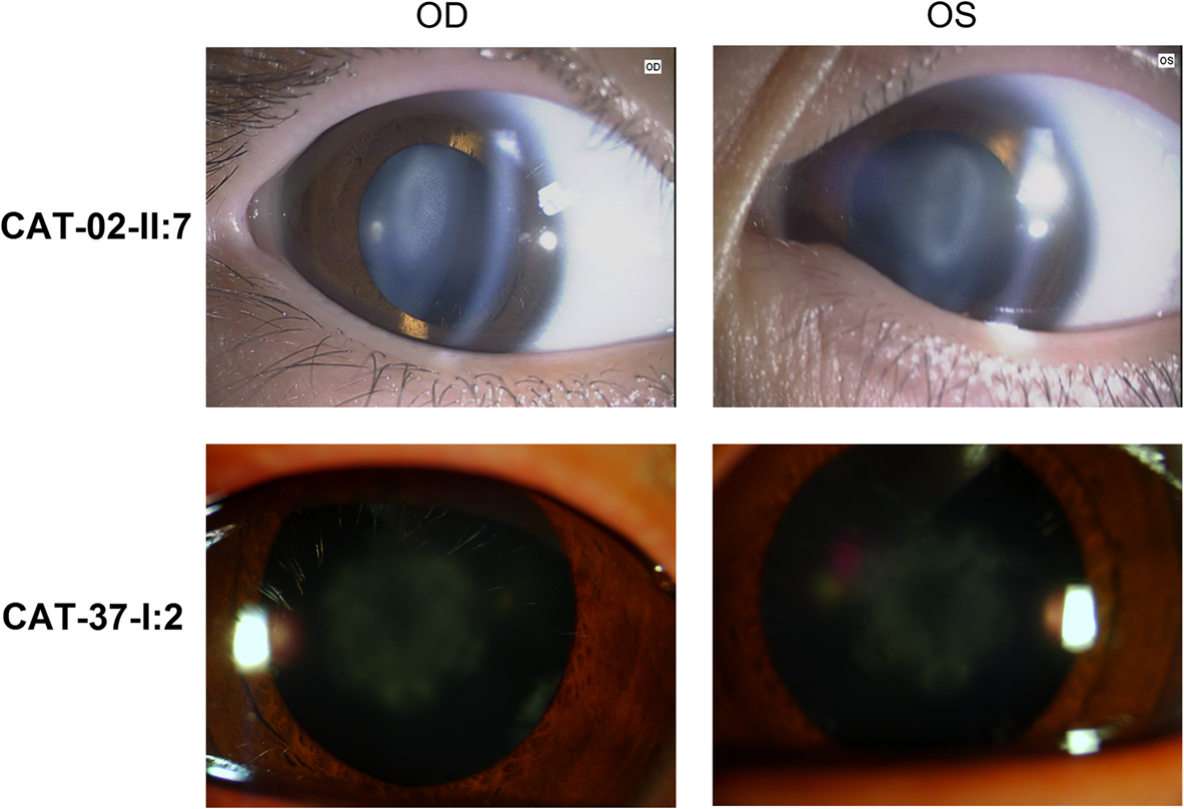
The slit-lamp photographs in two families. The slit-lamp photographs shows congenital nuclear cataract for CAT-37 family, and congenital perinuclear cataract for CAT-02 family

The 42 probands are composed of 30 probands from autosomal dominant families, 8 probands with no family history, and 4 isolated cases. In total, 379 individuals were recruited in this study. All the probands were diagnosed as bilateral cataracts at early childhood. No other ophthalmic or systemic diseases were found for all the patients. Five unrelated families were identified *HSF4* mutations. The inheritance pattern of those families is AD (Fig. 1). Based on clinical descriptions, family CAT-02 and family CAT-37 have been diagnosed as congenital perinuclear and nuclear cataracts, respectively`. Other three families including CAT-12, CAT-50 and CAT-51 with congenital total cataracts. The slit-lamp photographs of the patients in two families also showed the phenotype of cataracts (Fig. 2).

### Mutation analysis

**Fig. 3:**
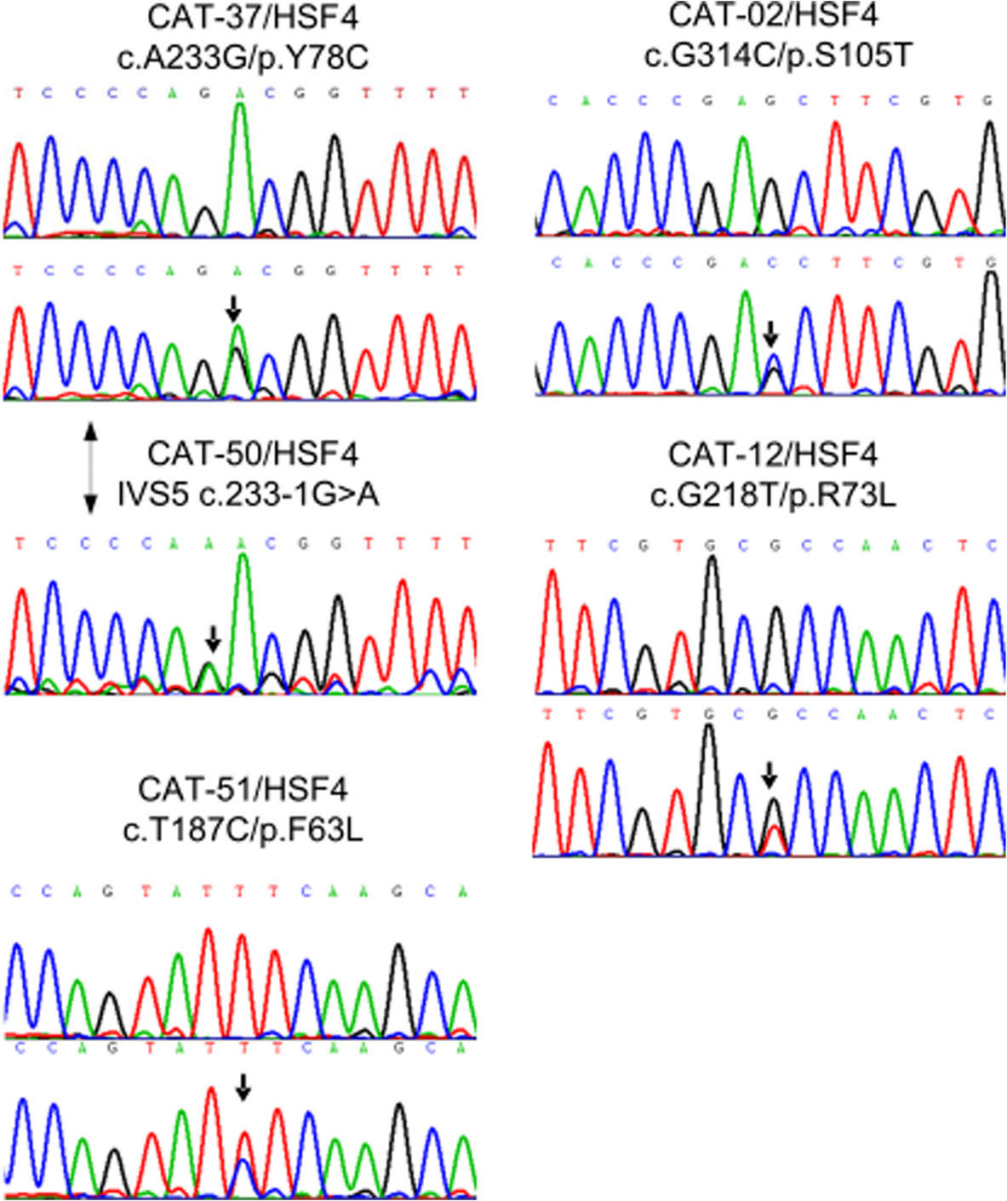
The 5 novel mutations in *HSF4* identified by direct sequencing. The black arrows indicate the mutations in the probands, and the wild type can be seen in the corresponding sequences from normal control

In total, five mutations were identified respectively using directly sequencing of the exons and flanking splicing sites of *HSF4* (Table 2 and Fig. 3), in five unrelated families with congenital cataracts. There were no variants on other seventeen causing genes detected in these five families. All of the five mutations have not been previously reported. Some mutations identified in other 17 families with congenital cataracts were not reported here. The clinical significances of the five mutations were generated by InterVar [9] software on the basis of the criteria recommended by ACMG/AMP guidelines. The process is automatically done firstly, and then follows a manual adjustment to reclassify the mutations for the criteria that InterVar recommends. These five mutations match the criterion of pathogenic moderate 1 (PM1) since all of them are in the hot spot regions. The five mutations were absent in the public databases including 1000 Genomes, ExAC and Genome Aggregation Database, which indicates they match the criterion of pathogenic moderate 2 (PM2). These five mutations were cosegregation with congenital cataracts in each affected family members (Figs. 1 and 3), indicating that they match the criterion of pathogenic supporting 1 (PP1).

**Table 2:** Classification of *HSF4* mutations in this study according to ACMG guideline

Family ID	Inheritance	Cataract Phenotype	Variation					PVS1	PM	PP			Classification
			gDNA change (hg19)	cDNA change	p.change	Status	type	PVS1	PM1	PM2	PP1	PP3	
CAT-02	AD	Perinuclear	g.67199703G > C	c.314G > C	p.Ser105Thr	Hetero	missense		Y	Y	*Y*	Y	Likely pathogenic
CAT-12	AD	Total	g.67199519G > T	c.218G > T	p.Arg73Leu	Hetero	missense		Y	Y	*Y*	Y	Likely pathogenic
CAT-37	AD	Nuclear	g.67199622A > G	c.233A > G	p.Tyr78Cys	Hetero	missense		Y	Y	*Y*	Y	Likely pathogenic
CAT-50	AD	Total	g.67199621G > A	IVS5 c.233-1G > A	/	Hetero	canonical splice sites	*Y*	*Y*	*Y*	*Y*	*Y*	Pathogenic
CAT-51	AD	Total	g.67199488 T > C	c.187 T > C	p.Phe63Leu	Hetero	missense		Y	Y	*Y*	Y	Likely pathogenic

*AD* autosomal dominant, *Hetero* heterozygosity, Manually adjustments (italic) were performed for the PP3 criteria for all the mutations and all the criteria for c.233-1G > A

PVS1 = The prevalence of the variant in affected individuals is significantly increased compared with the prevalence in controls;

PM1 = Located in a mutational hot spot and/or critical and well-established functional domain (e.g., active site of an enzyme) without benign variation;

PM2 = Absent from controls (or at extremely low frequency if recessive) in Exome Sequencing Project, 1000 Genomes Project, or Exome Aggregation Consortium;

PP1 = Cosegregation with disease in multiple affected family members in a gene definitively known to cause the disease;

PP3 = Multiple lines of computational evidence support a deleterious effect on the gene or gene product (conservation, evolutionary, splicing impact, etc.)

In Family CAT-02, the mutation was identified as a c.314G > C missense mutation, where a Serine was replaced by a Threonine at codon 105 (p.Ser105Thr). In Family CAT-12, a c.218G > T substitution was identified, which result in the replacement of an Arginine at position 73 by Leucine (p.Arg73Leu). In Family CAT-37, a c.233A > G missense mutation was identified, which led to Tyrosine at position 78 replaced by Cysteine (p.Tyr78Cys). In Family CAT-51, a c.187 T > C substitution led to Phenylalanine at position 63 replaced by Leucine (p.Phe63Leu). PolyPhen-2 predicted “probably damaging” of all the four variants except for c.233A > G (benign); while the SIFT method predicted “deleterious” for all the four variants except for c.314G > C (tolerated). These predictions indicated that the four variants may have effect on protein function. In Family CAT-50, a IVS5 c.233-1G > A mutation at 1 bp splice sites of the codon 78 is predicted to alter the WT acceptor site and most probably affect splicing by Human Splicing Finder software, which meet the criterion of pathogenic very strong (PVS1).

Finally, all five novel mutations were classified as “likely pathogenic” for congenital cataracts except for IVS5 c.233-1G > A mutation “pathogenic” using InterVar [9] software in accordance with ACMG standards. PM1, PM2 and PP3 were automated for the four missense mutations. The details of each mutation can be seen in the Table 2.

## Discussion

Heat-shock transcription factors (HSFs) activate heat-shock response genes under conditions of elevated temperature and other stress stimuli [10, 11]. All vertebrate HSFs share the property of lacking the carboxyl-terminal hydrophobic repeat. *HSF4,* a member of the HSF family, has been found to participate in the negative regulation of DNA binding activity [12]. Several studies presented that *HSF4* is required for ocular lens development and fiber cell differentiation [13–15]. Enoki Y et., al have showed that *HSF4* can activate transcription of genes encoding crystallins and beaded filament structural proteins in lens epithelial cells [16], whereas the mutations in *HSF4* gene may inhibit DNA-binding of *HSF4*, which may result in the loss of lens protein gene expression and cataractogenesis. Mutations in the human *HSF4* gene have been reported in both autosomal dominant and recessive cataracts.

**Fig. 4:**
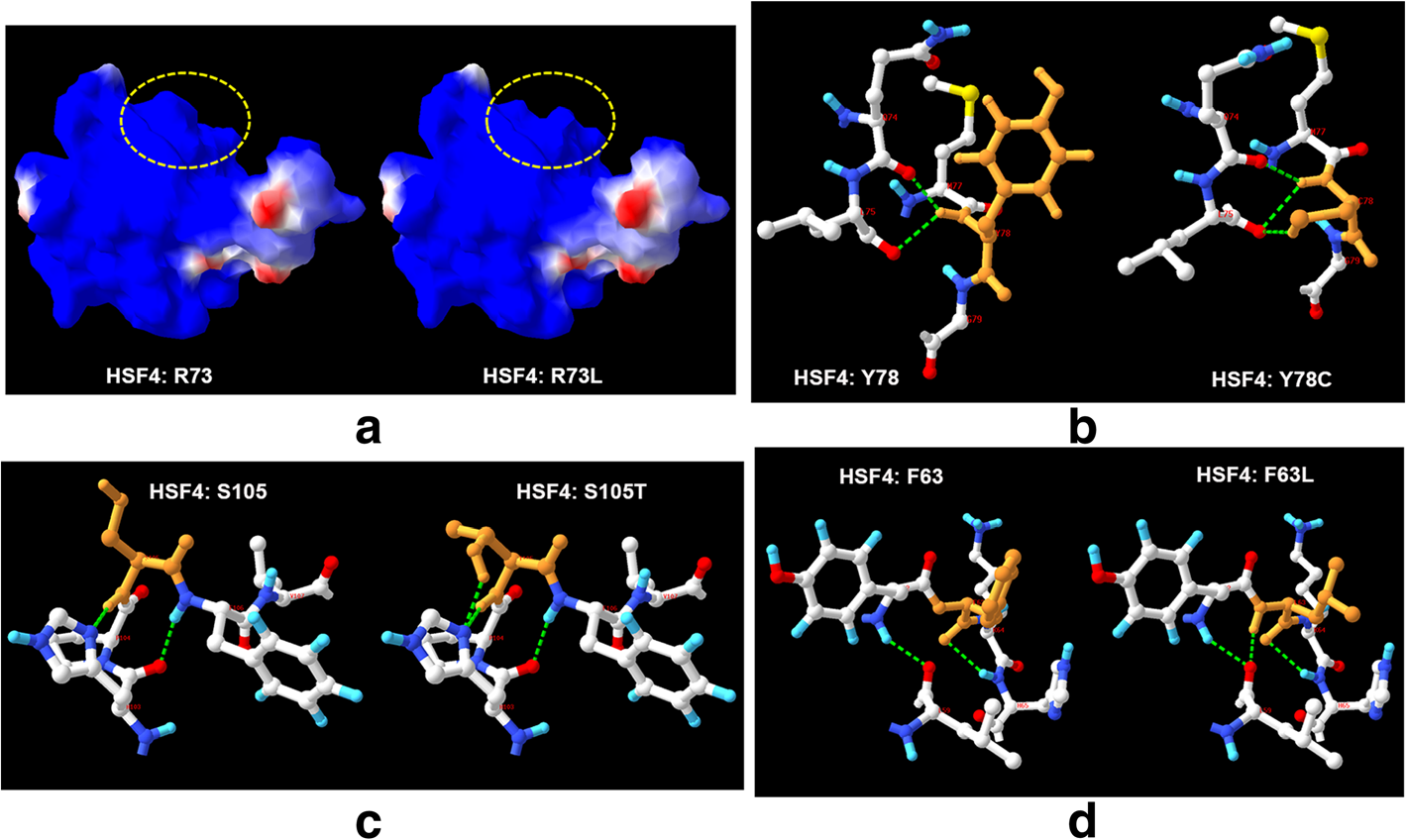
The structural changes of the missense mutations. **a** Surface change of *HSF4*:p.R73L. The molecular surface is colored according to electrostatic potential with Swiss-PdbViewer, with red-white-blue corresponding to acidic-neutral-basic potential. **b** *HSF4*:p.Y78C gains an H-bond with L75. **c** *HSF4*:p.S105 T gains an H-bond with P104. **d** *HSF4*:p.F63 L gains an H-bond with L59. Yellow dotted circle represents the region of significant alteration. Green dotted lines indicate potential strong H-bonds

In this study, we compiled hot-spot regions of cataract-causing mutations to sequence. All the five mutation reported here are in the hot-spot regions of cataract-causing mutations. Meanwhile, InterVar automatically reported that all the mutations match the criterion of pathogenic moderate 1 (PM1) because all of them are in the hot spot regions. The four missense mutations cause amino acid changes, which maybe eventually result in structural changes of the Heat-shock factor protein 4. Swiss-PdbViewer predicted that the molecular surface may be changed by R73L mutation, while an H-bond is probably obtained to connect Y78C with L75, S105 T with P104, and F63 L with L59, respectively (Fig. 4). The splicing mutation IVS5 c.233-1G > A, was predicted to be broken wild-type acceptor of *HSF4* protein, and most probably affecting splicing.

To date, ten mutations of *HSF4* gene have been reported to cause congenital cataract and additional two from ClinVar [17] (Table 3), including seven missense mutations (p.A19D, p.K23 N, p.R73H, p.I86V, p.L114P, p.L114P, p.R119C, p.R119C, p.R175P) [18–22], one nonsense mutations (p.R405X) [23], one splice mutation (c.1165-2A > G), and three frame shift mutations (p.D30Afs, p.G199EfsX15, p.M419GfsX29(delEx14) [24]).

**Table 3:** Summary of reported mutations in *HSF4* associated with congenital cataracts

Exon/Intron	DNAChange	CodingChange	Inheritance	Origin	Reference
Ex3	c.56C > A	p.A19D	AD	China	Bu et al. 2002[18]; ClinVar
Ex3	c.69G > T	p.K23 N	AD	China	Lv et al. 2014[19]
Ex3	c.89delA	p.D30Afs	/	US	ClinVar
Ex4	c.187 T > C	p.F63 L	AD	China	This study
Ex4	c.218G > A	p.R73H	AD	China	Ke et al. 2006[20]
Ex4	c.218G > T	p.R73L	AD	China	This study
IVS5	c.233-1G > A	/	AD	China	This study
Ex5	c.233A > G	p.Y78C	AD	China	This study
Ex5	c.256A > G	p.I86V	AD	China	Bu et al. 2002[18]; ClinVar
Ex5	c.314G > C	p.S105 T	AD	China	This study
Ex5	c.341 T > C	p.L114P	AD	China	Bu et al. 2002[18]; Clinvar
Ex5	c.341 T > C	p.L114P	AD	Denmark	Hansen et al. 2009[21]
Ex5	c.355C > T	p.R119C	AD	Denmark	Bu et al. 2002[18]; ClinVar
Ex5	c.355C > T	p.R119C	AD	Denmark	Hansen et al. 2009[21]
Ex7	c.524G > C	p.R175P	AR	Pakistan	Forshew et al. 2005[22]
Ex8	c.595-599del5bp	p.G199EfsX15	AR	Pakistan	Forshew et al. 2005[22]
IVS11	c.1165-2A > G	/	/	US	ClinVar
Ex12	c.1213C > T	p.R405X	AR	Pakistan	Sajjad et al. 2008[23]
IVS12	c.1327 + 4A > G	p.M419GfsX29(delEx14)	AR	Tunisia	Smaoui et al. 2004[24]

Together with five *HSF4* mutations reported here, Table 3 showed that *HSF4* mutations may be more requent in Chinese than in other world populations. These results suggest that direct screening *HSF4* mutations with one pair of primers, co-segregation and bioinformatics analyses, and mutation evaluation based on ACMG guidelines, might be a cost-effective, comprehensive and reliable method for molecular diagnosis of congenital cataracts.

## Conclusions

In summary, we identified five novel mutations in *HSF4* causing autosomal dominant congenital cataracts in Chinese families. *HSF4* mutations are responsible for 11.9% (five out of 42) of the families with congenital cataracts in our cohort. Our report extends the spectrum of *HSF4* mutations and may be helpful for the genetic diagnosis of congenital cataracts in the era of precision medicine.

## Additional file

Additional file 1: **Table S1**. The PCR primers and conditions for all the tested genes. The selected hot spot exons and splice junctions of these genes were amplified by PCR from genomic DNA using the primers and conditions. (DOCX 29 kb)

## Abbreviations

ACMG: American College of Medical Genetics and Genomics
AD: Autosomal dominant
AR: Autosomal recessive
CC: Congenital cataracts
HGVS: Human Genomic Variation Society
HSF: Human splicing finder v2.4.1
HSF4: Heat shock transcription factor 4
HSFs: Heat-shock transcription factors (HSFs)
PolyPhen-2: Polymorphism phenotyping v2
SIFT: Sorting intolerant from tolerant
