# Supplementary material for: Novel mutations in HSF4 cause congenital cataracts in Chinese families
Source: BMC Med Genet. 2018 Aug 24;19:150. doi: 10.1186/s12881-018-0636-3 (PMC6109319; doi:10.1186/s12881-018-0636-3)
Supplement: Supplementary file 1 — Table S1. The PCR primers and conditions for all the tested genes. The selected hot spot exons and splice junctions of these genes were amplified by PCR from genomic DNA using the primers and conditions. (DOCX 29 kb) [file 12881_2018_636_MOESM1_ESM.docx]

**Supplementary table 1 The PCR primers and conditions for all the tested genes**

| **Gene** | **Exon** | **Primer name** | Primer sequence（5'-3'） | **Amplicon size (bp)** | **PCR condition** |
| --- | --- | --- | --- | --- | --- |
| GJA8 (NM_005267) | 2 | GJA8e2F1 | TTGGAAAGGAGAGGTACCCC | 733 | 58℃ |
|  |  | GJA8e2R1 | CAGAGGCCACAGACAACATGA |  |  |
|  |  | GJA8e2F2 | TTCCGGATCCTGCCTCTGTA | 837 | 58℃ |
|  |  | GJA8e2R2 | CCTTTCATCTTGCCCTACGTA |  |  |
| GJA3 (NM_021954) | 2 | GAJ3E2-F1 | CCATCCCAGTACCATCCAG | 812 | 58℃/GC Buffer1 |
|  |  | GAJ3E2-R1 | CCTGCTTGAGCTTCTTCCA |  |  |
|  |  | GAJ3E2-F2 | ACGGTGGACTGCTTCATCTC | 830 | 58℃/GC Buffer1 |
|  |  | GAJ3E2-R2 | TCTATCTGCTGGTGGGAAGTG |  |  |
| CRYGD (NM_006891) | 1, 2 | CRYGDe1/2F | AGAACACGAAAATGCCCTTG | 579 | 58℃/GC Buffer1 |
|  |  | CRYGDe1/2R | TGCTTGAAACCATCCAGTGA |  |  |
| CRYAA (NM_000394) | 1 | CRYAAe1F | CTTCTTCATGAGCTCACGCC | 443 | 59℃ |
|  |  | CRYAAe1R | TGACGGAGCAAGACCAGAGT |  |  |
| CRYBB2 (NM_000496) | 6 | CRYBB2e6F3 | CTGACCCCAGTACAGTACAGT | 661 | 61℃ |
|  |  | CRYBB2e6R3 | CATTTCTCTCTCGCTGTCACTCTCTC |  |  |
| CRYBA1 (NM_005208) | 3 | CRYBA1e3F | ACTCTGGGCAAATGAACACC | 399 | 58℃ |
|  |  | CRYBA1e3R | TCCCCTATCCCCACTCTATG |  |  |
| CRYAA (NM_000394) | 3 | CRYAAe3F | CAGGCTAGGTCCAGAGAAG | 510 | 60℃/GC Buffer1 |
|  |  | CRYAAe3R | GGGAAGCAAAGGAAGACAGA |  |  |
| CRYAB (NM_001289807) | 3 | CRYABe3F | GTTGTCATGGCATTTGGTCTC | 433 | 58℃ |
|  |  | CRYABe3R | CTTGATAATTTGGGCCTGCC |  |  |
| CRYBA1 (NM_005208) | 4 | CRYBA1e4F | CCTGTCAACTCATTCCTCAACTC | 493 | 58℃ |
|  |  | CRYBA1e4R | CACCTGGTGGAGAAAAATCAA |  |  |
| CRYGD (NM_006891) | 3 | CRYGDe3F | CCTCACCAAGCTGGACTGC | 421 | 58℃ |
|  |  | CRYGDe3R | GCCAGGAACACACAGAAAATATT |  |  |
| CRYGC (NM_020989) | 3 | CRYGCe3F | CGCAGCAACCACAGTAATCT | 579 | 58℃ |
|  |  | CRYGCe3R | CCCACCCCATTCACTTCTTA |  |  |
| HSF4 (NM_001538) | 4, 5 | HSF4e4/5F | GGACCCAAGAGTGAGCATGA | 481 | 58℃/GC Buffer1 |
|  |  | HSF4e4/5R | CCCTCCTCCTCTTTGCTCAT |  |  |
| BFSP2 (NM_003571) | 3 | BFSP2e3F | CCCGGGAAGCCAGGTTATCAGAAGT | 566 | 58℃/GC Buffer1 |
|  |  | BFSP2e3R | TTTGAGACTGCTGGGGTAACCTGAC |  |  |
| EPHA2 (NM_004431) | 17 | EPHA2e17F | AGCTCTCTTGCCCTACAGGTCCCC | 498 | 59℃ |
|  |  | EPHA2e17R | CTAAGTGCTCAGCTGTGTGCGTCTC |  |  |
| PITX3 (NM_005029) | 4 | PITX3e4F | GCCACCTCATCTCGTTTATTG | 794 | 58℃/GC Buffer1 |
|  |  | PITX3e4R | GGGAGCAAGCCAGTCAAAA |  |  |
| FYCO1 (NM_024513) | 8 | FYCO1e8F-8 | AGCTGGGCATCCAGGTTT | 461 | 57℃ |
|  |  | FYCO1e8R-8 | CCTGCACCCACAGTACATTCT |  |  |
| CRYGC (NM_020989) | 1, 2 | CRYGCe1/2F | TGCATAAAATCCCCTTACCG | 556 | 58℃ |
|  |  | CRYGCe1/2R | CCTCCCTGTAACCCACATTG |  |  |
| CRYBB2 (NM_000496) | 5 | CRYBB2e5F | TGGGTGCACTGGGAAGAGA | 399 | 58℃/GC Buffer1 |
|  |  | CRYBB2e5R | GAAGCCAGAGGTCAGCAGAG |  |  |
| MIP (NM_012064) | 3 | MIPe3F | CCAGACAGGGCATCAGT | 373 | 58℃ |
|  |  | MIPe3R | TGGTACAGCAGCCAACAC |  |  |
| CRYBB1 (NM_001887) | 6 | CRYBB1e6F | GCACAGAGCAGGAAGGGATA | 498 | 58℃ |
|  |  | CRYBB1e6R | CGAGGAAGTCACATCCCAGT |  |  |
| CRYBB2 (NM_000496) | 2 | CRYBB2e2F | CCTTCAGCATCCTTTGGGTTCTCT | 597 | 58℃ |
|  |  | CRYBB2e2R | GCAGTTCTAAAAGCTTCATCAGTC |  |  |
| CRYGS (NM_017541) | 2 | [CRYGSe2F](http://www.ncbi.nlm.nih.gov/sites/entrez?Db=gene&Cmd=ShowDetailView&TermToSearch=1427&ordinalpos=7&itool=EntrezSystem2.PEntrez.Gene.Gene_ResultsPanel.Gene_RVDocSum) | GAAACCATCAATAGCGTCTAAATG | 229 | 58℃ |
|  |  | [CRYGSe2R](http://www.ncbi.nlm.nih.gov/sites/entrez?Db=gene&Cmd=ShowDetailView&TermToSearch=1427&ordinalpos=7&itool=EntrezSystem2.PEntrez.Gene.Gene_ResultsPanel.Gene_RVDocSum) | TGAAAAGCGGGTAGGCTAAA |  |  |
| MIP (NM_012064) | 1 | MIPe1F | GACTGTCCACCCAGACAAGG | 492 | 58℃ |
|  |  | MIPe1R | TCAGGGAGTCAGGGCAATAG |  |  |
| MIP (NM_012064) | 2 | MIPe2F | TGAAGGAGCACTGTTAGGAGATG | 500 | 58℃ |
|  |  | MIPe2R | AGAGGGATAGGGCAGAGTTGATT |  |  |
| CRYAB (NM_001289807) | 1 | CRYABe1F | AACCCCTGACATCACCATTC | 352 | 58℃ |
|  |  | CRYABe1R | AAGGACTCTCCCGTCCTAGC |  |  |
| CRYBA4 (NM_001886) | 4 | CRYBA4e4F | CTCCCCTAGTCGTGACAACC | 394 | 58℃ |
|  |  | CRYBA4e4R | TTTCAACTCTGGAACCTTTGA |  |  |
| CRYBB3 (NM_004076) | 6 | CRYBB3e6F | GAGGAATGTAGGCAGGCAGA | 480 | 58℃ |
|  |  | CRYBB3e6R | TCCTTCAGCACGCCTCTC |  |  |
